# HARDWIRED BIOLOGY AND LIGHT-BULB MOMENTS: DIVERGENT DISCOURSES AND LIFE TRAJECTORIES OF OLDER BISEXUAL WOMEN

**DOI:** 10.1093/geroni/igz038.3044

**Published:** 2019-11-08

**Authors:** Sarah Jen

**Affiliations:** University of Kansas School of Social Welfare, Lawrence, Kansas, United States

## Abstract

Older bisexual women report a less positive sense of their sexual identity, less belonging in LGBTQ communities, and worse mental health outcomes compared to lesbian counterparts. These patterns are consistent with those identified among younger bisexual cohorts and appear to be connected to how bisexual identities are perceived and experienced; however, sexual identities take on unique meaning by gender and age and across historical contexts. To explore how older bisexual women construct and make meaning out of bisexual identities, this study applied a Foucauldian discursive and critical feminist conceptual framing to examine semi-structured interviews with bisexual women ages 60 and older (N=13). Findings reveal two divergent groups of women, the Early Emergers and Mature Migrators, who differ in their constructions of bisexuality and the timing of their first experienced attractions to other women. While the Early Emergers construct bisexuality as a stable, “hardwired” biological concept, the Mature Migrators challenge this narrative by emphasizing the fluidity of sexuality through discourses of migration spurred by “light bulb” moments in which they first recognized their attractions to women. This study illustrates the contributions of discourse analysis in revealing nuanced constructions of life course histories as well as the need for acknowledgment of life context in research and practice with older bisexual individuals. Scholars and practitioners must intentionally critique and contribute to discourses of bisexuality in later life.

